# Tracing the HIV-1 subtype B mobility in Europe: a phylogeographic approach

**DOI:** 10.1186/1742-4690-6-49

**Published:** 2009-05-20

**Authors:** Dimitrios Paraskevis, Oliver Pybus, Gkikas Magiorkinis, Angelos Hatzakis, Annemarie MJ Wensing, David A van de Vijver, Jan Albert, Guiseppe Angarano, Birgitta Åsjö, Claudia Balotta, Enzo Boeri, Ricardo Camacho, Marie-Laure Chaix, Suzie Coughlan, Dominique Costagliola, Andrea De Luca, Carmen de Mendoza, Inge Derdelinckx, Zehava Grossman, Osama Hamouda, IM Hoepelman, Andrzej Horban, Klaus Korn, Claudia Kücherer, Thomas Leitner, Clive Loveday, Eilidh MacRae, I Maljkovic-Berry, Laurence Meyer, Claus Nielsen, Eline LM Op de Coul, Vidar Ormaasen, Luc Perrin, Elisabeth Puchhammer-Stöckl, Lidia Ruiz, Mika O Salminen, Jean-Claude Schmit, Rob Schuurman, Vincent Soriano, J Stanczak, Maja Stanojevic, Daniel Struck, Kristel Van Laethem, M Violin, Sabine Yerly, Maurizio Zazzi, Charles A Boucher, Anne-Mieke Vandamme

**Affiliations:** 1 Katholieke Universiteit Leuven, Rega Institute for Medical research, Minderbroederstraat 10, B-3000 Leuven, Belgium; 2 National Retrovirus Reference Center, Department of Hygiene Epidemiology and Medical Statistics, Medical School, University of Athens, M. Asias 75, GR-11527, Athens, Greece; 3 Department of Zoology, University of Oxford, South Parks Road, Oxford, OX1 3PS, UK; 4 University Medical Center Utrecht, Department of Virology, G04.614, Heidelberglaan 100, 3584 CX, Utrecht, the Netherlands; 5 Department of Virology, Erasmus MC, University Medical Centre, Postbus 2040 3000 CA Rotterdam, the Netherlands; 6 Department of Microbiology, Tumor and Cellbiology, Karolinska Institutet, SE 171 77 Stockholm, Sweden; 7 Dept of Virology, Swedish Institute for Infectious Disease Control, SE-171 82 Solna, Sweden; 8 University of Foggia, Clinic of Infectious Diseases, Ospedali Riuniti – Via L. Pinto 71100 Foggia, Italy; 9 Center for Research in Virology, University of Bergen, Bergen High Technology Center, N-5020 Bergen, Norway; 10 University of Milano, Institute of Infectious and Tropical Diseases, Via Festa del Perdono 7, 20122 Milano, Italy; 11 Diagnostica and Ricerca San Raffaele, Centro San Luigi, I.R.C.C.S. Istituto Scientifico San Raffaele, Milan, Italy; 12 Universidade Nova de Lisboa, Laboratorio de Virologia, Rua da Junqueira 96 1349-008 Lisboa, Portugal; 13 EA 3620, Universite Paris Descartes, Virologie, CHU Necker, Paris France; 14 National Virus Reference Laboratory, University College, Dublin, Ireland; 15 INSERM U263 et SC4, Faculté de médecine Saint-Antoine, Université Pierre et Marie Curie, 27 rue de Chaligny, F-75571 Paris, France; 16 Department of Infectious Diseases, Catholic University, L.go A. Gemelli, 8 00168 Rome, Italy; 17 Hospital Carlos III, Hospital Carlos III, Madrid, Spain; 18 Internal Medicine, UZ Leuven, Belgium; 19 National. HIV Reference Lab, Central Virology, Public Health Laboratories, MOH Central Virology, Sheba Medical Center, 2 Ben-Tabai Street, Israel; 20 Robert Koch Institut (RKI), Nordufer 20, 13353 Berlin, Germany; 21 University Medical Center Utrecht, Department of Internal Medicine and Infectious Diseases F02.126, Heidelberglaan 100, 3584 CX, Utrecht, the Netherlands; 22 Hospital for Infectious Diseases, Center for Diagnosis & Therapy Warsaw 37, Wolska Str. 01-201 Warszawa, Poland; 23 University of Erlangen, Schlossplatz 4, D-91054 Erlangen, Germany; 24 ICVC Charity Laboratories, 3d floor, Apollo Centre Desborough Road High Wycombe, Buckinghamshire, HP11 2QW, UK; 25 Inserm, U822, Le Kremlin-Bicêtre, F-94276, France; 26 Statens Serum Institut Copenhagen, Retrovirus Laboratory, department of virology, building 87, Division of Diagnostic Microbiology 5, Artillerivej 2300 Copenhagen, Denmark; 27 Centre for Infectious Disease Control (Epidemiology & Surveillance), National Institute for Public Health and the Environment (RIVM), 3720 BA Bilthoven, the Netherlands; 28 Ullevaal University Hospital, Department of Infectious Diseases Kirkeveien 166, N-0407 Oslo, Norway; 29 Laboratory of Virology, Geneva University Hospital and University of Geneva Medical School, Geneva, Switzerland; 30 Institute of Virology, Medical University Vienna, Kinderspitalgasse 15, Vienna, Austria; 31 IrsiCaixa Foundation, Hospital Germans Trias i Pujol, Ctra. de Canyet s/n, 08916 Badalona (Barcelona), Spain; 32 National Public Health Institute, HIV laboratory and department of infectious disease epidemiology, Mannerheimintie 166, FIN-00300 Helsinki, Finland; 33 Centre Hospitalier de Luxembourg, Retrovirology Laboratory, National service of Infectious Diseases, 4 Rue Barblé, L-1210, Luxembourg; 34 University of Belgrade School of Medicine, Institute of Microbiology and Immunology Virology Department, Dr Subotica 1, 11000 Belgrade, Serbia; 35 Section of Microbiology, Department of Molecular Biology, University of Siena, Italy

## Abstract

**Background:**

The prevalence and the origin of HIV-1 subtype B, the most prevalent circulating clade among the long-term residents in Europe, have been studied extensively. However the spatial diffusion of the epidemic from the perspective of the virus has not previously been traced.

**Results:**

In the current study we inferred the migration history of HIV-1 subtype B by way of a phylogeography of viral sequences sampled from 16 European countries and Israel. Migration events were inferred from viral phylogenies by character reconstruction using parsimony. With regard to the spatial dispersal of the HIV subtype B sequences across viral phylogenies, in most of the countries in Europe the epidemic was introduced by multiple sources and subsequently spread within local networks. Poland provides an exception where most of the infections were the result of a single point introduction. According to the significant migratory pathways, we show that there are considerable differences across Europe. Specifically, Greece, Portugal, Serbia and Spain, provide sources shedding HIV-1; Austria, Belgium and Luxembourg, on the other hand, are migratory targets, while for Denmark, Germany, Italy, Israel, Norway, the Netherlands, Sweden, Switzerland and the UK we inferred significant bidirectional migration. For Poland no significant migratory pathways were inferred.

**Conclusion:**

Subtype B phylogeographies provide a new insight about the geographical distribution of viral lineages, as well as the significant pathways of virus dispersal across Europe, suggesting that intervention strategies should also address tourists, travellers and migrants.

## Background

Pandemic HIV-1 group M infection originated in Africa from the simian immunodeficiency virus (SIVcpz) infecting chimpanzees [1-6]. The subtype B epidemic in the United States and elsewhere, was the result of a single point introduction -migration – of the virus from Haiti around the late sixties [7,8]. The introduction of HIV-1 into Europe occurred mainly through homosexual contacts or needle sharing in or from the USA [9-13], or through heterosexual contacts with individuals from Central Africa [14,15]. At the beginning of the HIV-1 epidemic (the early 1980's) the prevalence of HIV-1 infection was higher among men having sex with other men (MSM) than among heterosexuals. For this reason and also because subtype B was identified at a high prevalence among MSM in the USA, it was the predominant clade in Europe. The prevalence of non-B subtypes in Europe has been increasing over the last years [16-31]. However, the AIDS epidemic among the long-term residents is still dominated by viruses assigned to subtype B [32,33].

RNA viruses, such as the HIV-1, provide measurably evolving populations characterized by very high nucleotide substitution rate [34,35]. Phylogenies can be used for molecular epidemiology studies and notably they contain information about temporal and spatial dynamics of the virus [36]. The latter is the geographic pattern of viral lineages sampled from different localities, also termed as phylogeography, tracking the migration of the virus. For several viral infections, the dispersal of the parasite and its host cannot be easily tracked, therefore suggesting that phylogenies may be a better way to monitor migratory pathways of the virus [37,38]. This methodology has been recently applied to phylogeographic studies of influenza A (H5N1) [37] and HCV [39] epidemics showing the pathways of viral dispersal.

Thus, phylogenies are the 'state of the art' in characterizing viral genealogy and evolution and also serve as tools to track migration for organisms for which there is no other way to monitor their dispersal [38]. Although several phylogenetic studies have analyzed HIV-1 clades by geographic region in Europe, none has inferred the history of virus's migration through its phylogeny. In the present study, we inferred the migration history of HIV-1 virus among 17 countries in Europe, by way of a phylogeography of subtype B sequences.

## Results

**Figure 1:**
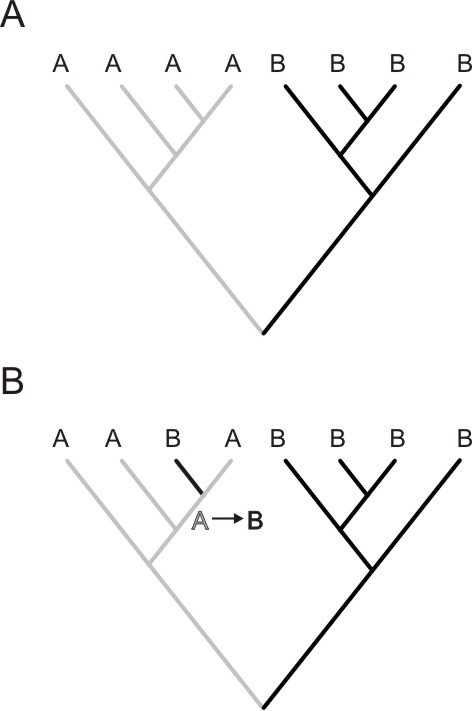
**This tree contains 8 sequences sampled from 2 countries (A and B)**. Tips (HIV-1 sequences) were labelled according to its sampling country. A. If there are no epidemiological links between the two populations A and B, viral sequences will consist of two monophyletic groups, therefore representing distinct epidemics. B. In case that an individual sampled within population B acquired the infection in geographic area A, one branch sampled from population B would cluster within the monophyletic clade of the population A. The migration pattern for each country was estimated by counting "state" (county label) changes at each internal node of the tree by the criterion of parsimony. For each country we counted "exporting" (From) and "importing" (To) migration events. Specifically, as shown in Fig. 1b, a state change (A-B) is counted as an exporting migration event for country A and as importing for B. In our study migration events correspond to mobility of HIV-1 strains or infections and, therefore, inferred exporting or importing migration events are proportional to country-wise mobility of HIV-1 subtype B strains.

Migration events were inferred through virus phylogenies by using the Slatkin and Maddison's method [40] (illustrated in Figure 1). Trees were built by maximum likelihood (ML) methodology and countries from which sequences were sampled were assigned to the tips of the 10^3 ^ML bootstrap trees. Inclusion of a large number of phylogenies takes into account phylogenetic uncertainty, because migration events are estimated over a set of trees rather than a single one.

### Phylogenetic analyses

**Figure 2:**
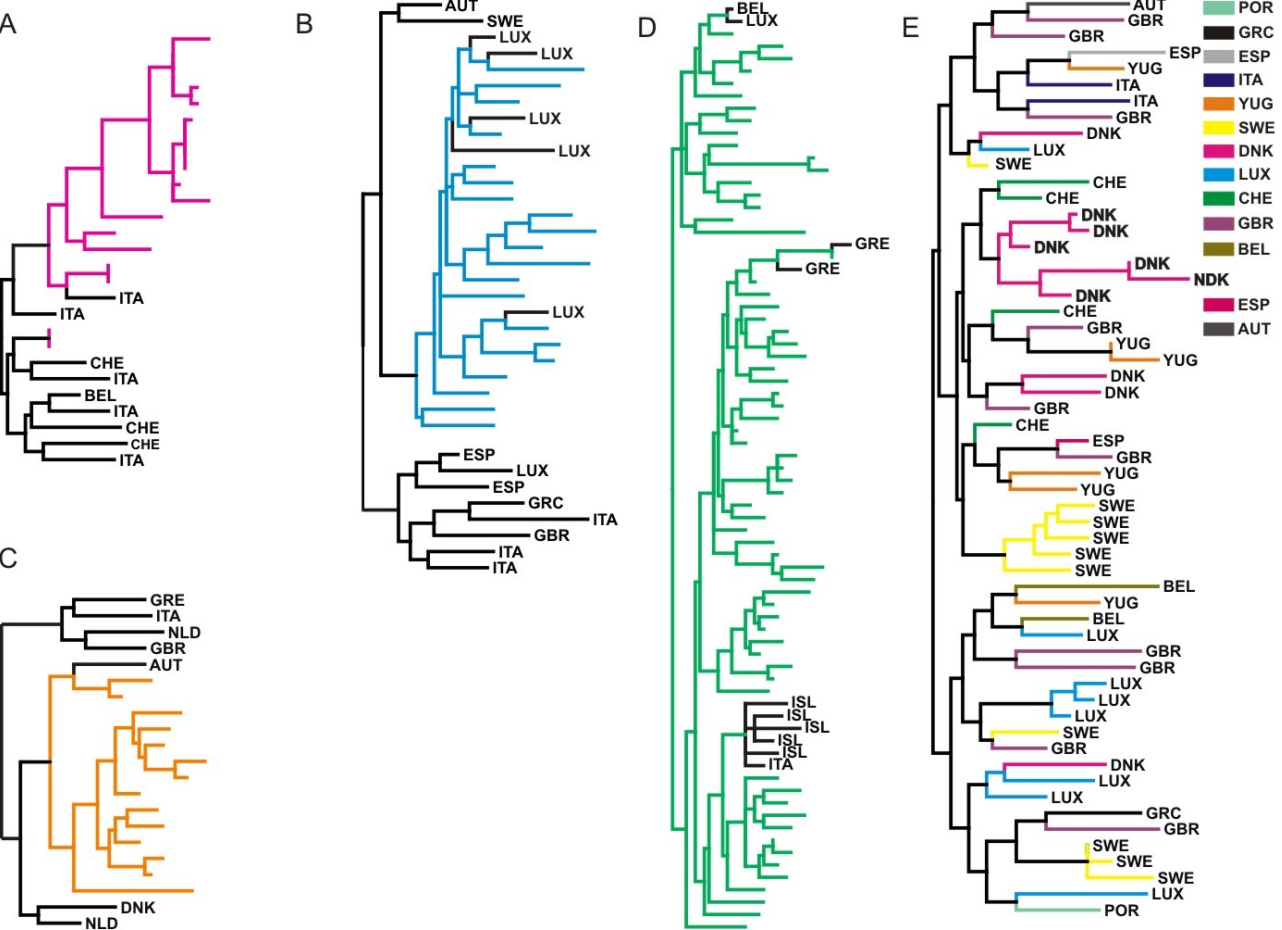
**Parts of the phylogenetic tree inferred for subtype B sequences sampled across Europe**. Monophyletic groups of sequences sampled from A. Austria (purple), B. Portugal (cyan), C. Luxembourg (orange) and D. Poland (green). E. Part of the tree showing the geographical dispersal of HIV-1 subtype B sequences. Branches are shown in different colours by country of origin as described in the legend. Branches are not drawn to scale.

Phylogenies of subtype B sequences from 16 countries in Europe and Israel (Table 1) showed no considerable grouping of sequences by country, however in the case of Poland most of the sequences (65, 72%) formed a single monophyletic clade (Figure 2). Similarly a fraction of sequences from Austria (16, 18%), Luxembourg (13, 14%) and Portugal (20, 22%) fell within single clusters, however the number of viral lineages spreading within local transmission networks was much lower in these areas than in Poland. Notably, in Poland individuals infected locally were mainly IDUs (39/65, 60%). Bayesian phylogenetic methods were used to further confirm the monophyletic nature of the B sequences from Poland, Austria, Luxembourg and Portugal. The final analysis was performed including a few sequences of the different monophyletic clusters identified in the ML trees and 1–2 from the other countries as references. Sequences again appeared as monophyletic in this analysis, with high posterior probability support (>0.8; data not shown), further supporting our previous results.

**Table 1:** Proportion of transmission risk groups among the study population.

	**Risk groups**					
	
**Country**	**MSM**	**IDUs**	**Heterosexuals**	**Others**	**Unknown**	**Sum**
United Kingdom (GBR)	59 (66%)	0 (0%)	6 (7%)	0 (0%)	25 (28%)	**90**
Austria (AUT)	18 (20%)	5(6%)	7 (8%)	0 (0%)	60 (67%)	**90**
Belgium (BEL)	56 (65%)	3 (3%)	11 (13%)	4 (5%)	12 (14%)	**86**
Denmark (DNK)	15 (17%)	4 (4%)	7 (8%)	0 (0%)	64 (71%)	**90**
Spain (ESP)	46 (51%)	21 (23%)	17 (19%)	0 (0%)	6 (7%)	**90**
Germany (DEU)	85 (94%)	0 (0%)	(0%)	0 (0%)	5 (6%)	**90**
Greece (GRC)	39 (53%)	3 (4%)	8 (11%)	1 (1%)	22 (30%)	**73**
Israel (ISR)	15 (44%)	8 (24%)	7 (21%)	1 (3%)	3 (9%)	**34**
Italy (ITA)	31 (34%)	15 (17%)	32 (36%)	0 (0%)	12 (13%)	**90**
Luxembourg (LUX)	50 (56%)	15 (17%)	19 (21%)	0 (0%)	6 (7%)	**90**
Netherlands (NLD)	57 (68%)	7 (8%)	15 (18%)	0 (0%)	5 (6%)	**84**
Norway (NOR)	19 (73%)	1 (4%)	5 (19%)	0 (0%)	1 (4%)	**26**
Poland (POL)	12 (13%)	42 (47%)	19 (21%)	0 (0%)	17 (19%)	**90**
Portugal (PRT)	27 (30%)	16 (18%)	35 (39%)	0 (0%)	12 (13%)	**90**
Serbia	22 (50%)	6 (14%	16 (36%)	0 (0%)	0 (0%)	**44**
Sweden (SWE)	44 (49%)	3 (3%)	10 (11%)	0 (0%)	33 (37%)	**90**
Switzerland (CHE)	48 (53%)	10 (11%)	28 (31%)	0 (0%)	4 (4%)	**90**

**Sum**	643 (48%)	159 (12%)	242 (18%)	6 (0.5%)	287 (21%)	**1337**

ML phylogenies suggest that sequences from the rest of Europe show distinct grouping patterns. Specifically a number of sequences for each locality cluster within short monophyletic clades (approximately consisting of 2–6 sequences), or others show no grouping according to their geographic origin (Figure 2E). These findings suggest that except in the case of Poland and also to a lesser extend for Austria, Portugal, Luxembourg, where a considerable percentage of infections were the result of single migration and subsequent spread among the local population, for the rest of countries there is a high level of mixing across Europe.

For patients recruited in the prospective study, information on the most likely origin of the HIV infection was collected through a questionnaire. Among them, 572 sequences were used in the current analysis. Interestingly, among those for whom this information was available (456 patients), 90.4% claimed that they acquired the subtype B.

### Statistical Phylogeography

**Figure 3:**
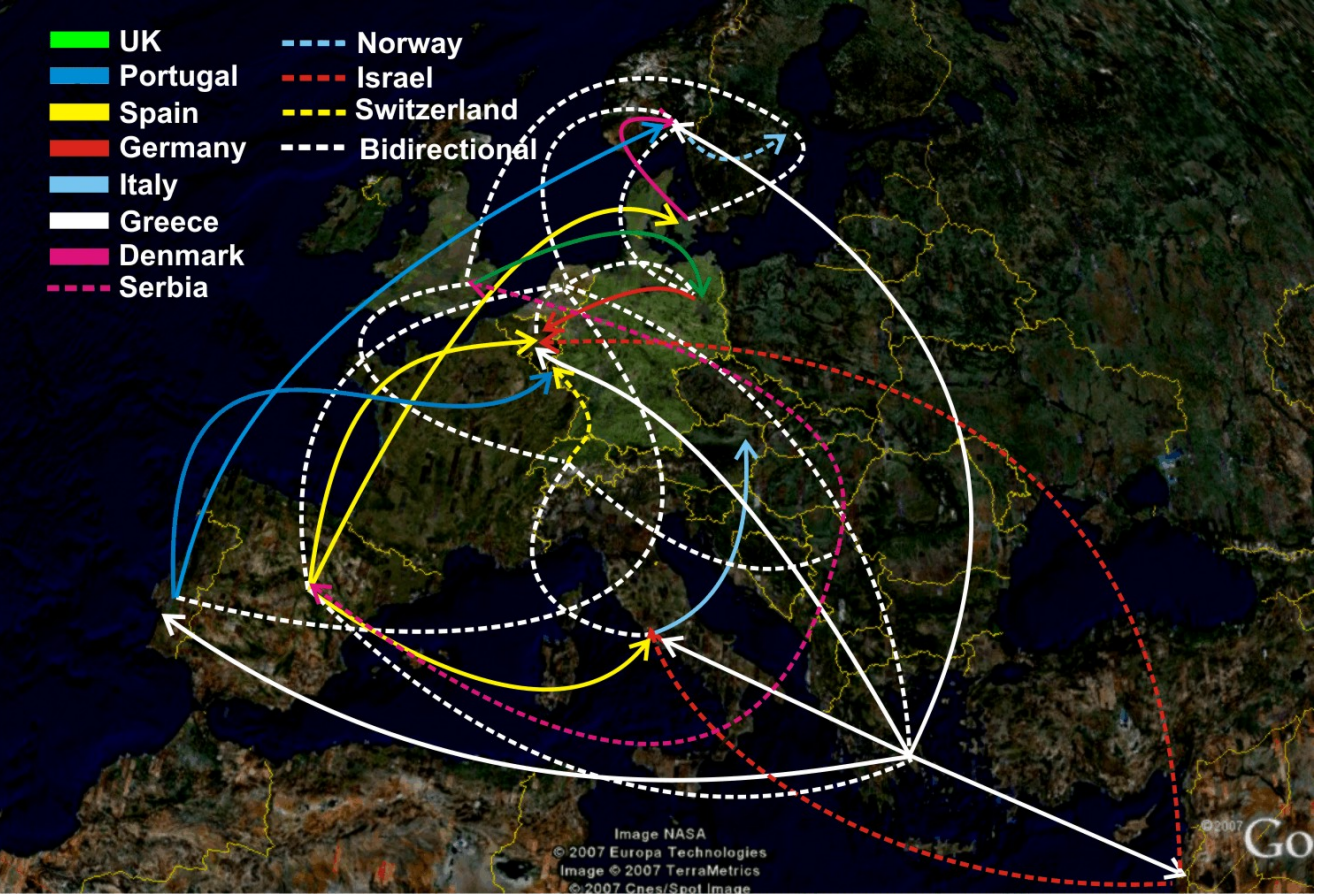
**Significant HIV migratory pathways across Europe**. Arrowheads indicate the targets of migration shown in different colours and styles by country of origin.

To test the significance of specific pathways of location changes (migration events) between countries, we estimated the expected number of changes, under the null hypothesis of complete geographic mixing, for each pair of countries (Tables S1 and S2 in Additional file 1), as described previously [37,39]. The total number of location changes between countries (migration events) for all trees was significantly lower than expected by chance under the null hypothesis of panmixis confirming that, although there is a high level of HIV dispersal between countries, there is still geographic subdivision among the subtype B lineages analyzed. Moreover, the results of this test showed major differences across Europe (Additional files 2 and 3). In particular, for Austria, Luxembourg and Poland no significant exporting migration was observed, while for the latter importing migration was also not significant; therefore classifying Poland as the country with the lowest HIV migration – or, in other words, with the most isolated HIV epidemic among the countries analysed (Figure 3). For Austria, and Luxembourg, on the other hand, there was evidence that some of the subtype B infections were the result of migration from Italy and Portugal, Switzerland, respectively; while similarly to Poland no significant outgoing migration was observed. According to the ML trees, only a few sequences from Israel and Greece fell within the Polish monophyletic cluster, suggesting limited migration to the latter countries (Figure 2D).

Germany, Greece, Italy, Norway, the Netherlands, Portugal, Spain, Serbia, Switzerland, and the UK appeared as source of subtype B mobility (high levels of exporting migration; "From") to other countries (Additional files 2 and 3). In case that significant migration was detected from a country to more than 2 others, the former was designated as "exporter". Notably, Greece's migratory targets were dispersed to 7 countries, while for both Spain and the Netherlands; they were to 5 and 6 countries, respectively (Figure 3). High levels of HIV migration – with regard to the highest difference between the observed and the expected migration events under panmixis – were detected from Italy to Austria and Switzerland, from Portugal to Luxembourg and also from the Netherlands to Germany (Table S2 in Additional file 1). On the other hand, Belgium, Denmark, Sweden and Israel showed only limited export of HIV-1 subtype B (Additional files 2 and 3).

Major migratory targets of HIV-1 subtype B (importing migration; "To") were Austria, Belgium, Germany, Italy, Luxembourg, Norway, the Netherlands, Sweden, Spain, Switzerland, and the UK (a similar criterion as for the "From" migration was used to assign countries) (Additional files 4 and 5), while limited migration was observed into Serbia and Israel (Supplementary information Figure 1c, d in Additional files 4 and 5) (in case that significant migration was detected from a country to more than 2 others, the former was designated as "exporter"). Notably, except from Poland, significant importing migration was detected for all countries across Europe (Figure 3).

Based on these findings, evidence for directional HIV dispersion was detected where Spain, Greece, Portugal and Serbia acted as sources of migration events ("exporters") (Figure 3); Austria, Belgium, and Luxembourg (Luxembourg and Austria were classified within the "importers" due to the high migration (>7) inferred from Portugal towards Luxembourg), provided migratory targets ("importers") (Figure 3), while significant bidirectional HIV migration was found for Denmark, Germany, Italy, Israel, Norway, the Netherlands, Sweden, Switzerland and the UK (Figure 3). Israel and Sweden were classified among localities with bidirectional migration because in both countries significant bidirectional mobility was detected. In contrast, for Poland, no significantly importing or exporting migration was found that is in accordance with the high percentage of sequences grouping according to the sampling location.

To further confirm our findings all steps of the analyses (phylogenetic analysis with ML bootstrapping, inference of migration events and statistical phylogeography) were repeated in a 2^nd ^run. Notably, migration events inferred on 10^3 ^newly inferred ML bootstrap trees were almost identical to the previous (R^2 ^= 0.98, *p *< 0.001; data not shown). Moreover, statistical phylogeography revealed that out of 46 and 50 significantly high migration events inferred in the two rounds of analyses, 43 were identical, thus suggesting that the major migratory pathways were reproducible.

## Discussion

Our results based on a phylogeographic study of a large number of sequences sampled from 16 countries in Europe and Israel provided important clues about HIV-1 subtype B spatial diffusion across Europe. Notably according to the findings of phylogenetic analyses, viral lineages sampled from all countries except Poland, Austria, Luxembourg and Portugal, showed low levels of grouping according to the geographic origin. For most countries, we identified small networks of local transmission, but to a different extent in each country, along with sequences showing no particular geographic clustering. Such a pattern suggests that the subtype B epidemic in most countries was introduced by several founders, some of them causing subsequent local dispersal, while others lead to dead end infections. We should note that under the conditions of our study, we cannot estimate the percentage of infections occurring within local transmission networks, since we don't have sufficient covering per country.

Poland's epidemic dispersal is quite different. Based on the high number of viral lineages coalescing to a common origin within the country, we suggest that the epidemic is the result of a few migrations of the virus successfully spreading within the local population. This pattern is consistent with a main viral dispersal through IDU networks associated with extensive local epidemics. Monophyletic HIV epidemics have been described among IDUs for other European countries, as well, including also non-B subtypes strains [12,13,26,27,42-45].

For Austria, Poland and Luxembourg we identified more extensive local transmission networks than for the other European countries. Similarly HIV local networks have been described for Canada, Greece and the UK [46-50].

According to the epidemiological data, most of the subtype B infections newly diagnosed during 2002–2004, occurred locally. The geographic distribution by means of the viral evolutionary history, the phylogeography, on the other hand, revealed high levels of viral dispersal. Both observations are not necessarily in contradiction. Rather, they suggest that most of the migration identified through phylogeography may date from earlier in the transmission chain, and that the pre-existing complexity of the epidemic (multiple sources of introduction from diverse localities) is the main reason for the continuous extensive geographical dispersal across the viral phylogeny. Particularly if there are multiple founders, subsequent infections will be dispersed, across the viral phylogeny, according to the geographic origin of the founders' source. This is in accordance with previous findings about multiple introductions of the subtype B infection through sexual intercourses or IDU across Europe [13,47,49,51,52].

In addition to epidemic dispersal patterns, our study provided important findings about HIV-1 subtype B major sources and targets for migratory events, as well as localities with bidirectional viral dispersion.

In particular, Greece, Portugal, and Spain attract many travellers and tourists, especially from Central Europe, thus suggesting that HIV dispersal from Southern to Central Europe may, at least in part, occur by travellers infected during their stay in Southern Europe .

For countries classified among the HIV migratory targets (Austria, Belgium and Luxembourg) the epidemic was mainly imported due to the high HIV mobility to these countries. According to the epidemiological information, the highest rate of imported infections from other European countries occurs in Luxembourg. Moreover, the fact that 13% of the population of Luxembourg is of Portuguese origin provides a plausible explanation for the migratory pathway from Portugal . Another significant pathway was tracked from Italy to Austria, in accordance with the high influx from Italy during recent years . Denmark provided migratory target from another Scandinavian country (Sweden) but also from Spain. This is in accordance with epidemiological findings that a percentage of HIV subtype B infections in Denmark originated from Sweden and Spain.

Additionally we identified several countries showing bidirectional migration. Notably, for the Netherlands 6 significant pathways were detected from and to the same localities. The Netherlands is among the countries in Europe with the most diverse geographical origin among newly diagnosed patients, confirmed by the high percentage of non-Dutch individuals among the newly HIV-infected patients during 2003–2004 [32,53]. Moreover, because of its policies, the Netherlands attracts foreign drug users and male homosexuals, two populations known to be at higher risk for HIV infection [51].

Migratory pathways inferred through viral phylogenies cannot be directly validated by other sources of information (epidemiological figures, mobility and immigration information, tourism, etc), because these data are not stratified by subtype. Moreover, due to the high mobility of population within Europe and the complexity of the epidemic spread, information about the locus of infection for an individual doesn't necessarily match with the geographic origin of the source. On the other hand, phylogenetic analysis of viral sequences provides a realistic approach for the reconstruction of HIV transmission chains or networks [36,46,47,49,54-56], therefore suggesting that statistical phylogeography is appropriate for inferring the spatial dispersal of a viral epidemic.

Given the high complexity of the epidemic, dense sampling is needed in order to accurately reconstruct the spatial characteristics of the subtype B infections in Europe. This provides one of the limitations of this study; on the other hand however the analysis of our dataset, which is the largest available at the time of analysis, provides for a first time a description of the geographic distribution of viral lineages as well as the significant migrations of HIV subtype B across Europe, by means of viral phylogenies. Dense sampling for each locality would be ideal for such purposes; however limited availability of sequences for several countries, as well as computation time provide as the major limitations for such a study.

We paid special attention to representativeness of our data. The prospective SPREAD collection strategy (data from 2002–2004) was specifically designed to avoid such a bias [53], while the retrospectively collected CATCH data (1996–2002) were sampled as part of national surveillance studies designed to investigate the transmission of drug resistance or as part of the standard clinical practice of baseline sequencing for all newly diagnosed cases in each participating center [57]. For most countries where national data were available, the data were a rather good representation of the national epidemic.

In conclusion, HIV-1 subtype B phylogeographies provide a new insight for the first time into the pathways of spatial diffusion and virus migration across Europe. HIV-1 subtype B was each time introduced from multiple sources and subsequently spread locally, but the pattern is not uniform across Europe. The countries grouped into sources (Greece, Portugal, Serbia and Spain) and sinks (Austria, Belgium and Luxembourg) of virus migration, as well as countries with significant bidirectional migration (Denmark, Germany, Italy, Israel, Norway, the Netherlands, Sweden, Switzerland and the UK). The only exception was Poland where a significant number of sequences fell within a monophyletic cluster. These results suggest that mobility of the virus matches mobility of the host, such that in order to reduce further spread of the epidemic, prevention measures should not only be directed towards national populations, but also towards migrants, travellers and tourists who are the major sources and targets of HIV dispersal.

## Methods

### HIV-1 sequences

Protease (PR) and partial reverse transcriptase (RT) sequences were sampled from HIV-1 seropositive individuals who had never received antiretroviral drugs (ARV) as described previously [53,57]. Specifically, partial PR/RT sequences were sampled from 17 countries in Europe including Israel. Sequences were collected from two studies, the Combined Analysis of Resistance Transmission over Time of Chronically and Acute Infected HIV Patients; (CATCH), in a retrospective setting [57] and a prospective study named after Strategy to Control SPREAD of HIV Drug Resistance (SPREAD) [53]. In the CATCH analysis all sequences were collected during 1996–2002 from geographically distinct centres across the participating countries, except for Belgium and the Netherlands, where HIV-1 sequences were sampled from a single geographic area. In the prospective setting (SPREAD), samples were collected during 2002–2004 according to two different approaches in order to ensure representative sampling [53]. Notably although data from the period 1996–2002 were retrospectively analyzed, they were collected as part of national surveillance studies designed to investigate the transmission of drug resistance or of the standard clinical practice of baseline sequencing for all newly diagnosed cases in each participating center [57]. In the prospective setting a standardized sampling strategy was designed in order to ensure representative sampling in all countries [53]. For the purpose of this study we included only those classified as subtype B. All individuals were sampled at a single time point. The subtyping process was performed by phylogenetic analysis [53,57]. The prevalence of the transmission risk groups among the study population is shown in Table 1.

### Phylogenetic analyses

#### Sampling strategy

For the estimation of country-wise clustering (migration), first we need to infer the phylogenies of the sequences under study. One of the issues to be addressed was how many sequences needed to be included for each country. The dataset size needs to be large enough as: 1) to include most of the available information from each country and 2) to estimate rare migration events. On the other hand, we had to restrict the number of sequences to keep the computation time needed for phylogenetic inference reasonable, while maintaining an informative number of sequences required for the calculation of migration events. For this reason, we performed a preliminary analysis of migration for 4 countries including 10, 20 25 or 90 sequences per country. For each dataset, we tested whether the distribution of the total number of migration events across the set of all credible trees differed significantly from a distribution of randomly generated trees (phylogenetic inference was performed by ML method). The results of this preliminary analysis showed that with 25 sequences per country, the largest number of countries reached significantly different migration levels than compared to the distribution for a random set of trees (*P *< 0.01). However the larger the number of sequences included per country the higher the signal for clustering with regard the total number of changes across inferred versus random set of trees.

Consequently, we included in the analyses the largest number of sequences (90) available per country, expect from Belgium, Greece, the Netherlands, Israel, Norway and Serbia for which a smaller number of sequences was available, however only for the last three countries the number of sequences included was << 90. As a result of choosing approximately equal number of strains per country, irrespective of the prevalence or the total number of infected individuals across Europe, we calculated the relative mobility per infected individual. Therefore, the numbers in the migration matrices are directly comparable reflecting actual differences in mobility between countries. For example, we estimated higher migration from the UK to Spain (5.34), than from Germany to Italy (3.23) (Table S2 in Additional file 1).

Phylogenetic analyses for the estimation of the migration process were performed in a single dataset consisting of 1337 sequences analyzed in two independent runs (Table 1).

#### Alignment and phylogenetic tree reconstruction

The alignment of the subtype B partial RT sequences sampled from 1337 individuals was performed using CLUSTAL W version 1.74 [58] and manually edited according to the encoded reading frame. In order to avoid any bias due to convergent evolution at antiretroviral drug resistance mutations on the phylogenetic analysis, we excluded all sites associated with major resistance in PR (30, 32, 33, 46, 47, 48, 50, 54, 76, 82, 84, 88, and 90) and RT (41, 62, 65, 67, 69, 70, 74, 75, 77, 100, 103, 106,108, 115, 116, 151, 181, 184, 188, 190, 210, 215, 219, 225, and 236) leaving 687 nt.

Phylogenetic trees were inferred by maximum likelihood method under the general time-reversible GTR model of nucleotide substitution including a Γ distributed rates heterogeneity among sites as implemented in RAxML [59]. Bootstrapping was performed on the maximum likelihood trees (1000 replicates) to assess the reliability of the obtained topologies.

#### Inference of migration events

All bootstrap generated trees (10^3^) were used for the estimation of the HIV-1 migration events by using the cladistic approach first described by Slatkin and Maddison [40], as implemented in MacClade [60]. Specifically, all the nodes of the inferred trees were assigned with a character according to the geographic origin (e.g. 0, 1, 2, 3 for Austria, Belgium, Denmark, France, etc). The algorithm reconstructs "ancestral" states that in our case correspond to countries, at each internal node by the criterion of parsimony [40]. Parsimony selects the reconstruction that minimizes the total number of steps on the tree [41].

When two branches from 2 different locations (e.g. 0 and 1) join with each other, and thus more than one character can be reconstructed at the node, then the ancestor state at the internal node is assigned to be the union of the two characters [0, 1] that is assigned a migration event. If this number between two groups of sequences remains low, the possibility for migration events between these particular groups also remains low.

Specifically, the migration events between HIV-1 sequences sampled in different locations were estimated for each dataset according to the following method: 1) for nodes with more than one equally parsimonious reconstructions (e.g. 0, 1 or 0), implicit examination of all most parsimonious reconstructions (MPRs) was used in case of a big number of MPRs [61,62], while explicit examination was used in case of a small number of MPR, as implemented in MacClade. As a result, for a particular type of character change, e.g. [0,1] MacClade reports a minimum, a maximum and a average number of [0,1] changes estimated over all possible MPRs. We estimated the average number of migration events for each tree used in the analyses. 2) Polytomies that correspond to nodes with more than two descendant nodes were interpreted as regions of uncertain evolution (soft polytomies) as implemented in MacClade.

#### Inference of migration matrices

For each dataset a 17 × 17 migration matrix was estimated between HIV-1 sequences sampled in different European countries. Each migration event was calculated as the median of the distribution estimated from all trees (10^3^) used in the analysis. In the matrix, all 'from' events and 'to' events are pooled per country.

#### Statistical phylogeography

To further estimate which migration events were significantly different from the expected number of changes under the null hypothesis of full geographic mixing of HIV-1 sequences, we estimated if the distribution for each of the migration events estimated over 10^3 ^bootstrap trees was statistically different from the distribution estimated from the same set of trees (10^3^) after reshuffling taxa at the tips. This analysis was performed using Mesquite [63]. Equality of medians between observed and expected migration events was assessed by means of the Kruskal-Wallis one-way analysis of variance and the level of significance was adjusted according to Bonferroni correction for multiple comparisons.

The differences between the observed and the expected values indicate the levels of HIV-1 country-dependent structure in the dataset, and thus also of the relative mobility of the virus between countries. This strategy allowed estimating significant differences also when an unequal number of strains were included per country.

Notably in order to assess the validity of our results, the whole process of phylogenetic analysis, inference of migration events and statistical phylogeography was repeated twice.

## Competing interests

The authors declare that they have no competing interests.

## Authors' contributions

DP designed the study performed the analysis and prepared the manuscript, OP, GM and AH designed part of the analysis, AMJW and DAV collected the data and coordinated CATCH and SPREAD studies, JA, GA, BÅ, CB, EB, RC, MLC, SC, DC, ADL, CDM, ID, ZG, OH, IMH, AH, KK, CK, TL, CL, EMR, IM, LM, CN, ELMO, VO, VO, LP, EPS, LR, MS, JCS, RS, VS, JS, MS, DS, KVL, MV, SY, and MZ provided their data (protease and partial reverse transcriptase HIV-1 sequences together with epidemiological data). CAB coordinated CATCH and SPREAD-studies and AMV designed the study. All authors contributed to writing the paper

## Supplementary Material

Additional file 1 **Tables S1 and S2**. Table S1 – Number of calculated migration events (medians) between countries. Table S2 – Differences of the medians between observed and the expected migration events. Cells in bold and underlined bold denote significantly higher and lower migration numbers, respectively. Click here for file

Additional file 2 **Figure S1 (part A)**. Significant HIV exporting (**A **and **B**) and importing (**C **and **D**) migration events between different countries as estimated by statistical phylogeography study For all countries, 90 sequences were included per analysis, except for Belgium (BEL), Greece (GRC) and the Netherlands (NLD) for which 86, 73 and 84 sequences were included. For Israel (ISR), Norway (NOR) and Serbia (YUG) <<< 90 sequences were available, respectively. This lower number of sequences explains why the significantly high migration count for these countries is lower than for the other countries. Country code as in table 1. Click here for file

Additional file 3 **Figure S1 (part B)**. Significant HIV exporting (A and B) and importing (C and D) migration events between different countries as estimated by statistical phylogeography study For all countries, 90 sequences were included per analysis, except for Belgium (BEL), Greece (GRC) and the Netherlands (NLD) for which 86, 73 and 84 sequences were included. For Israel (ISR), Norway (NOR) and Serbia (YUG) <<< 90 sequences were available, respectively. This lower number of sequences explains why the significantly high migration count for these countries is lower than for the other countries. Country code as in table 1. Click here for file

Additional file 4 **Figure S1 (part C)**. Significant HIV exporting (A and B) and importing (C and D) migration events between different countries as estimated by statistical phylogeography study For all countries, 90 sequences were included per analysis, except for Belgium (BEL), Greece (GRC) and the Netherlands (NLD) for which 86, 73 and 84 sequences were included. For Israel (ISR), Norway (NOR) and Serbia (YUG) <<< 90 sequences were available, respectively. This lower number of sequences explains why the significantly high migration count for these countries is lower than for the other countries. Country code as in table 1. Click here for file

Additional file 5 **Figure S1 (part D)**. Significant HIV exporting (A and B) and importing (C and D) migration events between different countries as estimated by statistical phylogeography study For all countries, 90 sequences were included per analysis, except for Belgium (BEL), Greece (GRC) and the Netherlands (NLD) for which 86, 73 and 84 sequences were included. For Israel (ISR), Norway (NOR) and Serbia (YUG) <<< 90 sequences were available, respectively. This lower number of sequences explains why the significantly high migration count for these countries is lower than for the other countries. Country code as in table 1. Click here for file
