# Supplementary material for: Tracing the HIV-1 subtype B mobility in Europe: a phylogeographic approach
Source: Retrovirology. 2009 May 20;6:49. doi: 10.1186/1742-4690-6-49 (PMC2717046; doi:10.1186/1742-4690-6-49)
Supplement: Additional file 1 — Tables S1 and S2. Table S1 – Number of calculated migration events (medians) between countries. Table S2 – Differences of the medians between observed and the expected migration events. Cells in bold and underlined bold denote significantly higher and lower migration numbers, respectively. [file 1742-4690-6-49-S1.doc]

***Supplementary information*** ***Table 1*** *Number of calculated migration events (medians) between countries*

| **To**  **From** | **GBR** | **AUT** | **BEL** | **DNK** | **ESP** | **DEU** | **GRC** | **ISR** | **ITA** | **LUX** | **NLD** | **NOR** | **POL** | **PRT** | **SWE** | **CHE** | **YUG** |
| --- | --- | --- | --- | --- | --- | --- | --- | --- | --- | --- | --- | --- | --- | --- | --- | --- | --- |
| **GBR** |  | 5.03 | 4.85 | 4.87 | 5.34 | 5.88 | 2.53 | 1.05 | 4.47 | 5.25 | 3.38 | 1.16 | 1.42 | 2.70 | 6.68 | 5.45 | 2.60 |
| **AUT** | 2.95 |  | 2.59 | 2.73 | 2.35 | 2.53 | 1.58 | 1.24 | 4.78 | 2.58 | 2.90 | 0.63 | 0.45 | 2.53 | 2.81 | 2.97 | 0.78 |
| **BEL** | 3.47 | 3.02 |  | 3.31 | 3.26 | 4.02 | 2.72 | 0.97 | 3.14 | 3.88 | 4.71 | 1.42 | 1.09 | 3.21 | 3.04 | 2.94 | 0.83 |
| **DNK** | 4.14 | 3.42 | 3.91 |  | 4.19 | 4.72 | 2.47 | 1.20 | 4.03 | 3.11 | 3.44 | 3.07 | 1.39 | 2.95 | 6.38 | 4.17 | 1.87 |
| **ESP** | 4.94 | 4.27 | 5.69 | 5.35 |  | 5.17 | 5.16 | 1.64 | 5.93 | 4.21 | 5.50 | 1.67 | 1.56 | 4.78 | 5.26 | 4.68 | 2.42 |
| **DEU** | 5.21 | 4.42 | 5.60 | 5.30 | 4.63 |  | 3.63 | 1.49 | 3.23 | 2.59 | 5.70 | 2.12 | 2.36 | 3.86 | 3.94 | 3.59 | 0.82 |
| **GRC** | 2.87 | 2.38 | 3.58 | 2.95 | 4.32 | 3.40 |  | 2.04 | 3.71 | 1.96 | 4.00 | 1.52 | 1.52 | 3.92 | 3.00 | 3.16 | 0.13 |
| **ISR** | 0.42 | 0.57 | 1.35 | 0.58 | 0.50 | 0.46 | 0.66 |  | 2.32 | 0.28 | 0.46 | 0.12 | 0.38 | 0.59 | 0.42 | 0.63 | 0.00 |
| **ITA** | 3.80 | 8.21 | 4.57 | 5.05 | 5.11 | 2.72 | 2.49 | 1.70 |  | 4.94 | 4.32 | 0.92 | 0.76 | 3.29 | 3.37 | 6.75 | 1.96 |
| **LUX** | 3.80 | 2.56 | 3.45 | 2.74 | 3.09 | 2.18 | 1.03 | 0.33 | 3.02 |  | 2.41 | 0.33 | 0.67 | 1.94 | 3.12 | 4.40 | 1.98 |
| **NLD** | 3.80 | 3.70 | 5.51 | 4.40 | 5.08 | 6.42 | 4.50 | 1.48 | 4.15 | 3.03 |  | 2.04 | 2.09 | 5.15 | 3.72 | 2.58 | 0.30 |
| **NOR** | 0.44 | 0.39 | 0.60 | 0.58 | 0.52 | 0.75 | 0.52 | 0.12 | 0.48 | 0.30 | 0.63 |  | 0.30 | 0.68 | 0.83 | 0.33 | 0.00 |
| **POL** | 0.60 | 0.37 | 1.19 | 0.62 | 0.66 | 1.01 | 2.32 | 1.50 | 0.67 | 1.26 | 0.87 | 0.36 |  | 0.71 | 0.45 | 0.34 | 0.01 |
| **PRT** | 2.88 | 3.08 | 4.42 | 3.95 | 4.57 | 3.76 | 4.34 | 1.54 | 3.60 | 7.25 | 5.08 | 2.06 | 1.42 |  | 3.49 | 2.50 | 0.16 |
| **SWE** | 5.46 | 3.22 | 3.60 | 5.68 | 4.14 | 3.48 | 2.23 | 0.93 | 3.42 | 3.53 | 2.67 | 1.44 | 0.94 | 2.36 |  | 4.09 | 1.88 |
| **CHE** | 5.83 | 4.26 | 4.94 | 5.18 | 4.71 | 3.81 | 1.33 | 1.67 | 5.99 | 5.32 | 1.78 | 0.42 | 1.08 | 1.74 | 5.16 |  | 3.83 |
| **YUG** | 1.89 | 1.34 | 0.80 | 1.33 | 1.52 | 0.74 | 0.04 | 0.00 | 1.14 | 1.21 | 0.13 | 0.00 | 0.02 | 0.08 | 1.16 | 1.85 |  |

*For all countries, per dataset, 90 sequences were sampled except for Belgium (BEL), Greece (GRC) and the Netherlands (NLD) for which 86, 73 and 84 sequences were included. For Israel (ISR), Norway (NOR) and Serbia (YUG) <<< 90 sequences were available, respectively. Migration events are thus directly comparable except for ISR, NOR and YUG. To obtain total migration events per country, the results need to be scaled according to the number of infections per country.*

***Supplementary information Table 2*** *Differences of the medians between observed and the expected migration events. Cells in bold and italic bold denote significantly higher and lower migration numbers, respectively*

| **To**  **From** | **GBR** | **AUT** | **BEL** | **DNK** | **ESP** | **DEU** | **GRC** | **ISR** | **ITA** | **LUX** | **NLD** | **NOR** | **POL** | **PRT** | **SWE** | **CHE** | **YUG** |
| --- | --- | --- | --- | --- | --- | --- | --- | --- | --- | --- | --- | --- | --- | --- | --- | --- | --- |
| **GBR** |  | -0.13 | ***-0.26*** | ***-0.29*** | 0.15 | **0.76** | ***-1.80*** | ***-1.12*** | ***-0.57*** | 0.22 | ***-1.59*** | ***-0.45*** | ***-3.73*** | ***-2.37*** | **1.49** | **0.17** | ***-0.14*** |
| **AUT** | ***-2.17*** |  | ***-2.41*** | ***-2.34*** | ***-2.69*** | ***-2.49*** | ***-2.75*** | ***-0.81*** | ***-0.26*** | ***-2.46*** | ***-1.90*** | ***-1.03*** | ***-4.61*** | ***-2.44*** | ***-2.33*** | ***-2.12*** | ***-1.98*** |
| **BEL** | ***-1.22*** | ***-1.71*** |  | ***-1.26*** | ***-1.45*** | ***-0.71*** | ***-1.28*** | ***-0.91*** | ***-1.43*** | ***-0.85*** | **0.36** | -0.04 | ***-3.53*** | ***-1.40*** | ***-1.71*** | ***-1.63*** | ***-1.67*** |
| **DNK** | ***-1.00*** | ***-1.77*** | ***-1.15*** |  | ***-0.95*** | ***-0.54*** | ***-1.75*** | ***-0.94*** | ***-1.14*** | ***-2.08*** | ***-1.61*** | **1.41** | ***-3.79*** | ***-2.16*** | **1.25** | ***-1.01*** | ***-0.88*** |
| **ESP** | ***-0.05*** | -0.85 | **0.88** | **0.25** |  | 0.04 | **0.81** | ***-0.62*** | **0.83** | ***-1.00*** | **0.48** | 0.01 | ***-3.63*** | ***-0.35*** | -0.03 | ***-0.47*** | ***-0.41*** |
| **DEU** | 0.06 | ***-0.85*** | **0.52** | 0.16 | ***-0.50*** |  | ***-0.90*** | ***-0.66*** | ***-1.95*** | ***-2.62*** | **0.83** | **0.40** | ***-2.83*** | ***-1.49*** | ***-1.16*** | ***-1.69*** | ***-1.97*** |
| **GRC** | ***-0.47*** | ***-0.99*** | **0.30** | ***-0.42*** | **0.92** | 0.02 |  | **0.64** | **0.52** | ***-1.29*** | **0.84** | **0.44** | ***-1.84*** | **0.63** | ***-0.25*** | ***-0.13*** | ***-1.68*** |
| **ISR** | ***-0.48*** | ***-0.37*** | **0.45** | ***-0.34*** | ***-0.46*** | ***-0.42*** | ***-0.12*** |  | **1.39** | ***-0.61*** | ***-0.39*** | ***-0.17*** | ***-0.52*** | ***-0.30*** | ***-0.50*** | ***-0.27*** | ***-0.49*** |
| **ITA** | ***-1.25*** | **3.13** | ***-0.17*** | 0.02 | 0.10 | ***-2.27*** | ***-1.82*** | ***-0.39*** |  | -0.10 | ***-0.57*** | ***-0.70*** | ***-4.25*** | ***-1.74*** | ***-1.63*** | **1.66** | ***-0.77*** |
| **LUX** | ***-1.33*** | ***-2.64*** | ***-1.42*** | ***-2.26*** | ***-2.09*** | ***-2.97*** | ***-3.21*** | ***-1.90*** | ***-2.05*** |  | ***-2.51*** | ***-1.31*** | ***-4.41*** | ***-3.20*** | ***-2.08*** | ***-0.63*** | ***-0.79*** |
| **NLD** | ***-0.60*** | ***-0.71*** | **1.32** | 0.07 | **0.71** | **2.00** | **0.78** | ***-0.35*** | -0.19 | ***-1.27*** |  | **0.69** | ***-2.25*** | **0.79** | ***-0.66*** | ***-1.75*** | ***-2.07*** |
| **NOR** | ***-0.20*** | ***-0.24*** | 0.05 | -0.01 | ***-0.09*** | **0.15** | 0.00 | ***-0.11*** | ***-0.13*** | ***-0.34*** | **0.05** |  | ***-0.31*** | 0.04 | **0.25** | ***-0.30*** | ***-0.37*** |
| **POL** | ***-4.51*** | ***-4.68*** | ***-3.86*** | ***-4.59*** | ***-4.51*** | ***-3.99*** | ***-2.04*** | ***-0.61*** | ***-4.48*** | ***-3.74*** | ***-4.01*** | ***-1.28*** |  | ***-4.41*** | ***-4.59*** | ***-4.84*** | ***-2.77*** |
| **PRT** | ***-2.31*** | ***-1.89*** | ***-0.50*** | ***-1.05*** | ***-0.37*** | ***-1.41*** | 0.06 | ***-0.56*** | ***-1.46*** | **2.14** | **0.27** | **0.34** | ***-3.78*** |  | ***-1.63*** | ***-2.64*** | ***-2.60*** |
| **SWE** | **0.32** | ***-1.89*** | ***-1.40*** | **0.50** | ***-1.06*** | ***-1.61*** | ***-2.21*** | ***-1.20*** | ***-1.81*** | ***-1.67*** | ***-2.13*** | ***-0.16*** | ***-4.21*** | ***-2.85*** |  | ***-0.96*** | ***-0.82*** |
| **CHE** | **0.79** | ***-0.89*** | -0.02 | 0.00 | ***-0.38*** | ***-1.25*** | ***-2.99*** | ***-0.49*** | **0.92** | **0.20** | ***-3.10*** | ***-1.16*** | ***-4.11*** | ***-3.56*** | 0.10 |  | **1.01** |
| **YUG** | **0.47** | ***-0.04*** | ***-0.51*** | -0.07 | **0.13** | ***-0.62*** | ***-1.20*** | ***-0.61*** | ***-0.20*** | ***-0.15*** | ***-1.18*** | ***-0.41*** | ***-1.42*** | ***-1.33*** | ***-0.23*** | **0.47** |  |

*Negative values indicate less mobility than expected by chance, whereas positive values indicate more mobility than expected. For Israel (ISR), Norway (NOR) and Serbia (YUG) <<< 90 strains were included resulting in a lower number of migration events to be expected. In such cases, the panmixis hypothesis test allows to estimate whether or not a significantly higher (or lower) number of migration events were observed.*
