# Supplementary figures and images for: Tracing the HIV-1 subtype B mobility in Europe: a phylogeographic approach
Source: Retrovirology. 2009 May 20;6:49. doi: 10.1186/1742-4690-6-49 (PMC2717046; doi:10.1186/1742-4690-6-49)

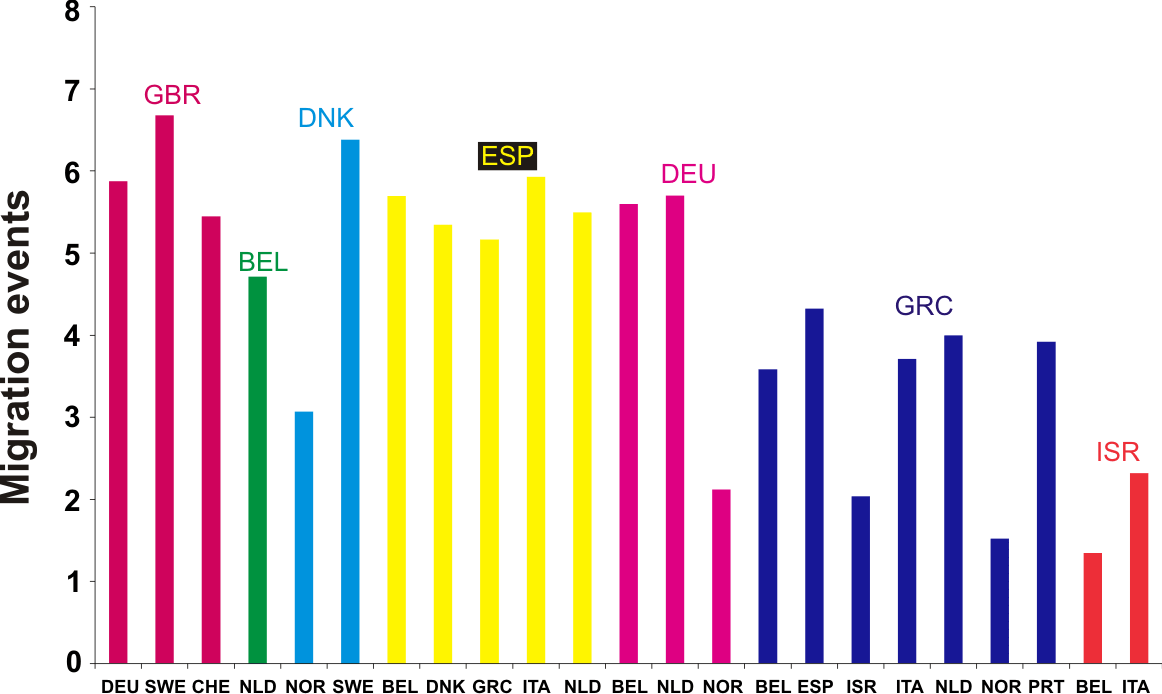

Supplement: Additional file 2 — Figure S1 (part A). Significant HIV exporting (A and B) and importing (C and D) migration events between different countries as estimated by statistical phylogeography study For all countries, 90 sequences were included per analysis, except for Belgium (BEL), Greece (GRC) and the Netherlands (NLD) for which 86, 73 and 84 sequences were included. For Israel (ISR), Norway (NOR) and Serbia (YUG) <<< 90 sequences were available, respectively. This lower number of sequences explains why the significantly high migration count for these countries is lower than for the other countries. Country code as in table 1. [file 1742-4690-6-49-S2.gif]

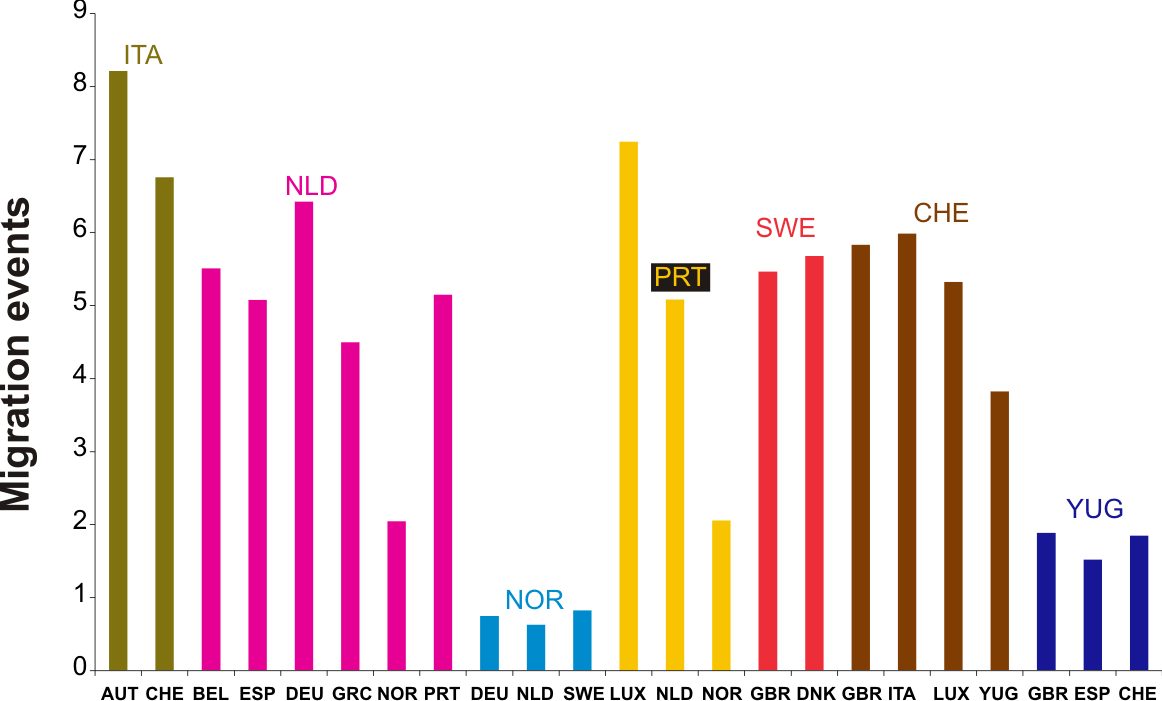

Supplement: Additional file 3 — Figure S1 (part B). Significant HIV exporting (A and B) and importing (C and D) migration events between different countries as estimated by statistical phylogeography study For all countries, 90 sequences were included per analysis, except for Belgium (BEL), Greece (GRC) and the Netherlands (NLD) for which 86, 73 and 84 sequences were included. For Israel (ISR), Norway (NOR) and Serbia (YUG) <<< 90 sequences were available, respectively. This lower number of sequences explains why the significantly high migration count for these countries is lower than for the other countries. Country code as in table 1. [file 1742-4690-6-49-S3.gif]

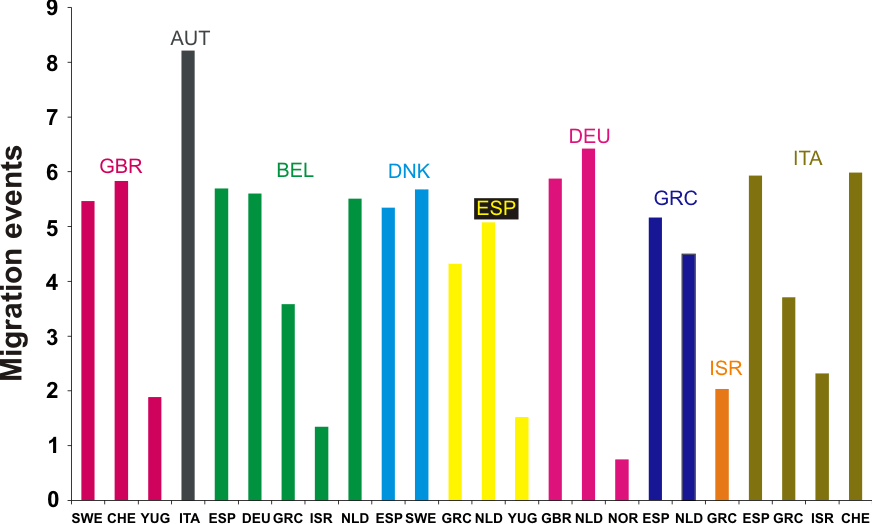

Supplement: Additional file 4 — Figure S1 (part C). Significant HIV exporting (A and B) and importing (C and D) migration events between different countries as estimated by statistical phylogeography study For all countries, 90 sequences were included per analysis, except for Belgium (BEL), Greece (GRC) and the Netherlands (NLD) for which 86, 73 and 84 sequences were included. For Israel (ISR), Norway (NOR) and Serbia (YUG) <<< 90 sequences were available, respectively. This lower number of sequences explains why the significantly high migration count for these countries is lower than for the other countries. Country code as in table 1. [file 1742-4690-6-49-S4.gif]

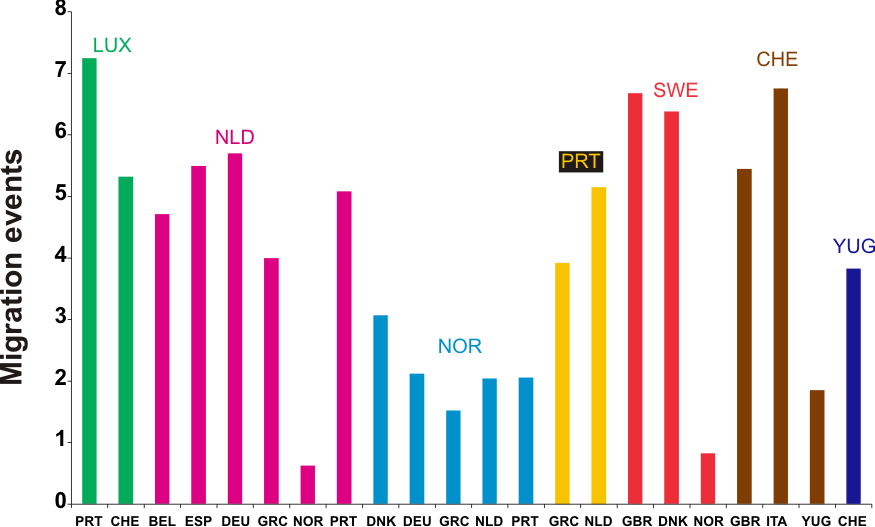

Supplement: Additional file 5 — Figure S1 (part D). Significant HIV exporting (A and B) and importing (C and D) migration events between different countries as estimated by statistical phylogeography study For all countries, 90 sequences were included per analysis, except for Belgium (BEL), Greece (GRC) and the Netherlands (NLD) for which 86, 73 and 84 sequences were included. For Israel (ISR), Norway (NOR) and Serbia (YUG) <<< 90 sequences were available, respectively. This lower number of sequences explains why the significantly high migration count for these countries is lower than for the other countries. Country code as in table 1. [file 1742-4690-6-49-S5.gif]
